# Editorial: Altered bioenergetics, mitochondrial quality control, and calcium signaling in the brain: Implications to age-related diseases

**DOI:** 10.3389/fragi.2023.1171221

**Published:** 2023-03-01

**Authors:** Smijin K. Soman, Bridget Martinez, Ruben K. Dagda

**Affiliations:** Department of Pharmacology, University of Nevada, Reno School of Medicine, Reno, NV, United States

**Keywords:** mitochondria, diabetes mellitus, retinal degeneration, 2-deoxyglucose, d-Glyceric acid

## Background

The human body has the innate capacity to undergo limited tissue regeneration and maintain homeostasis to prolong longevity. However, several factors contribute to age-related decline in cellular function and the onset of aging-related diseases. Changes in the epigenetic imprint of the human genome and disruption in critical signaling pathways that regulate proteostasis, biogenesis, and the recycling and lysosomal turnover of organelles are among the factors that contribute to age-related changes and diseases. Beyond their classical role in generating ATP, the energy currency that powers essential cellular functions, mitochondria are “multi-tasking” organelles that serve as hubs to regulate cell homeostasis, including the intake and efflux of excess calcium, produce intracellular reactive oxygen species (ROS) that can serve as second messengers to activate downstream signaling pathways, act as heat generators by decoupling ATP production, enable the synthesis of a variety of lipids and cholesterol, and modulate cell survival. Recent studies have highlighted the importance of mitochondria as hubs and critical platforms by which nuclear and cytosolic signaling pathways and cell membrane receptors (e.g., Estrogen receptor and epidermal growth factor receptor) can converge at the outer mitochondrial membrane to uncouple cell proliferation and modulate protein homeostasis, quality control pathways, calcium signaling, and stress response pathways. To this end, normal mitochondrial function is critical for maintaining cell homeostasis and health. Indeed, a disruption in mitochondrial function and structure in affected tissues leads to altered calcium homeostasis, an increase in mitochondrial-derived ROS, and eventual disruption of energy production (oxidative phosphorylation + glycolysis). At the cellular level, an increase in ROS disrupts critical biogenesis and turnover signaling pathways by oxidizing signaling macromolecules leading to cell death by activating apoptosis or necrosis. At the organ system level, mitochondrial dysfunction can contribute to cognitive decline, neurodegeneration, and motor symptoms in neurodegenerative diseases like Parkinson’s disease, altered insulin signaling and metabolism leading to diseases such as type 2 diabetes through increased insulin resistance, and finally, can also increase the metastatic potential of tumors. However, recent advances in the biomedical sciences have led to the development of treatment regimens or therapies in the pipeline that can improve patient care by enhancing mitochondrial function. The current research topic, which includes one brief research report and three original research articles from renowned investigators, showcases new knowledge regarding underlying pathological factors that contribute to age-related decline in tissues and the characterization of new treatments (e.g., antioxidants) and therapies that have the potential to increase the quality of life in patients and longevity in age-related diseases.

### Synopsis of research articles on the special topic

1) Hirvonen et al., found that a 4-day exogenous administration of the small molecule dihydroxyacetone (DGA) resulted in significant changes in various biomarkers related to energy metabolism, including lactate, beta-hydroxybutyrate (bHB), and creatine kinase (CK). These changes suggest an increase in whole-body mitochondrial activation and cellular maintenance reactions, as well as improvements in hepatic activation and reductions in subclinical inflammation. The authors hypothesize that the effects are caused by an indirect signal or cascade of intracellular signals triggered by an increase in tissue and cellular DGA concentration, which they named “DGA activation.” The study was limited to participants with normal weight and the effects carried on longer than expected. Future research should investigate both short- and long-term effects of the DGA activation, including its potential benefits for aging populations.
2) Dodson et al., examined the effects of glucose deprivation and 2-deoxy-D-glucose (2DG) on cell viability, mitochondrial function, and the effects of the neurotoxic lipid hydroxynenal (HNE) on neuronal survival and mitochondrial function in primary rat cortical neurons. In brief, the results showed that, in the presence of HNE, 2DG caused a greater decrease in cell viability and exacerbated HNE-induced mitochondrial dysfunction more than glucose deprivation in primary neurons. Additionally, glucose deprivation had less impact on HNE-induced mitochondrial dysfunction than 2DG. The study also found that changing glucose availability altered HNE-induced damage of mitochondrial electron transport chain complexes I and II. Overall, the findings suggest that 2DG and glucose deprivation have distinct effects on neuronal mitochondria, which could have important implications for neurodegenerative diseases.
3) Peng et al., explored the protective effects of the antioxidant epicatechin (EC) on retinal degeneration in a mouse model of dry age-related macular degeneration (AMD). The study aimed to investigate the potential therapeutic benefits of EC on retinal degeneration and explore its molecular mechanisms of action by using *in vivo* and *in vitro* models. In brief, the study found that EC protected the retina against NaIO3-induced degeneration, reduced drusen-like deposits, and maintained mitochondrial morphology in retinal pigment epithelial cells. The study also identified key genes and pathways associated with AMD by employing gene expression profiling data of human retina samples.
4) Wu et al., reported significant changes in the brain-related networks of patients with type 2 diabetes mellitus (T2DM) compared to healthy individuals. In brief, the study conducted independent component analysis (ICA) and functional connectivity analysis on the brain networks of the participants. The results showed that T2DM patients had significant differences in hemoglobin A1c levels, BMI, MoCA-B scores, and grooved pegboard (R) compared to the healthy group. In addition, the analysis of the brain networks showed that T2DM patients had high positive connectivity between the salience network (SN) with the primary visual network (PVN) and the left executive control network (LECN) with the extrastriate visual network (EVN). Negative connectivity was observed between the right executive control network (RECN) with the SN, default mode network (DMN), PVN, EVN, and LECN. The T2DM patients also showed differences in the internal and external network connectivity, and dynamic functional network connectivity compared to the healthy group. State 3 and state 4 were significantly different in the T2DM group. Overall, connectivity in the language network (LN), precuneus network, EVN, and PVN increased, whereas LECN, EVN, and PVN connectivity was lower in the T2DM group compared with the NC group.

